# Rare Case Report: Left Atrial Sarcoma Obstructing the Left Ventricular Inflow

**DOI:** 10.3390/jcm12206460

**Published:** 2023-10-11

**Authors:** Ann-Sophie Kaemmerer, Mathieu N. Suleiman, Abbas Agaimy, Frank Harig, Michael Weyand, René Tandler

**Affiliations:** 1 Department of Cardiac Surgery, University Hospital Erlangen, Friedrich-Alexander-University Erlangen-Nürnberg, 91054 Erlangen, Germany; mathieu.suleiman@uk-erlangen.de (M.N.S.); frank.harig@uk-erlangen.de (F.H.); michael.weyand@uk-erlangen.de (M.W.); rene.tandler@uk-erlangen.de (R.T.); 2 Institute of Pathology, University Hospital Erlangen, Friedrich-Alexander-University Erlangen-Nürnberg, 91054 Erlangen, Germany; abbas.agaimy@uk-erlangen.de

**Keywords:** undifferentiated pleomorphic cardiac sarcoma, cardiac surgery, malignant cardiac tumor, left atrial tumor, cardiac sarcoma

## Abstract

Malignant cardiac tumors of the heart are extremely rare and may present tremendous diagnostic and therapeutic challenges. These tumors are able to infiltrate the heart and metastasize systemically. Early detection is often elusive as the clinical presentation is highly variable, posing significant diagnostic and therapeutic difficulties. Despite a multidisciplinary approach, the prognosis for patients with malignant cardiac tumors remains guarded. Early diagnosis and a multidisciplinary approach involving cardiac surgeons, oncologists and critical care specialists are crucial in the management of this disease. Further research is needed to better understand the pathomechanisms of tumor-related complications and to develop effective treatment strategies to improve patient outcomes. The rare case of a 78-year-old woman with left atrial tumor requiring emergency surgery for acutely developing mitral valve obstruction is presented. Pathology confirmed an undifferentiated pleomorphic sarcoma. This patient tragically did not survive, highlighting the difficulties of managing such a rare and deceptive heart disease.

## 1. Introduction

Primary tumors of the heart are rare and often benign [1]. They can be found within the heart, on the valves or even in the pericardium. They vary widely in their characteristics, origins and clinical implications. Primary malignant cardiac tumors are very rare, originating from various cell types within the heart. They usually are undifferentiated (intimal) sarcomas, including angiosarcomas, rhabodomyosarcomas and fibrosarcomas, which have the capability to locally infiltrate cardiac structures, interfere with cardiac function, as in the presented case and metastasize to distant sites [1]. The diversity of their clinical features adds to the complexity of diagnosis and treatment and requires a multidisciplinary approach. The clinical presentation of malignant cardiac tumors is highly variable, often mimicking other cardiac or noncardiac conditions. In some cases, these tumors remain completely asymptomatic until they reach an advanced stage. Common but atypical symptoms include dyspnea, palpitations, chest pain and signs of heart failure [2,3,4,5]. Because of their varied presentation and rarity, they are often misdiagnosed or remain undetected until later stages.

Imaging modalities like echocardiography, CT and MRI play a crucial role in the detection of these tumors [6]. However, the gold standard for definitive diagnosis remains histopathologic analysis of biopsy material or surgically removed tissue [7,8]. 

Surgical resection is often the primary treatment, but due to the anatomic location and potential for metastasis, achieving complete excision can be challenging [9]. Definitive treatment requires a multimodal approach combining surgery, chemotherapy, radiotherapy and, in selected cases, targeted therapies or immunotherapies. However, malignant cardiac tumors carry a serious prognosis because they are often refractory to any therapy [3,10]. 

This case is of particular significance due to the infrequency of cardiac sarcomas and the complex diagnostic and therapeutic intricacies it entails. The tumor exhibited an initial presentation that mimicked a myxoma, falsely suggesting benignity. The true malignancy only came to light upon pathological examination. Consequently, this case stands out for its unforeseen diagnostic complexity and underscores the formidable challenges encountered when distinguishing between benign and malignant cardiac masses within intricate clinical contexts.

### Case Report

A 78-year-old diabetic woman (158 cm, 60.9 kg, BMI: 24.4 kg/m^2^) was referred from a regional hospital where she presented with worsening general condition, increasing dyspnea, atrial fibrillation and a history of a thromboembolic cerebral event. Chest X-ray showed a markedly enlarged heart, pulmonary venous congestion and large bilateral pleural effusions.

**Figure 1:**
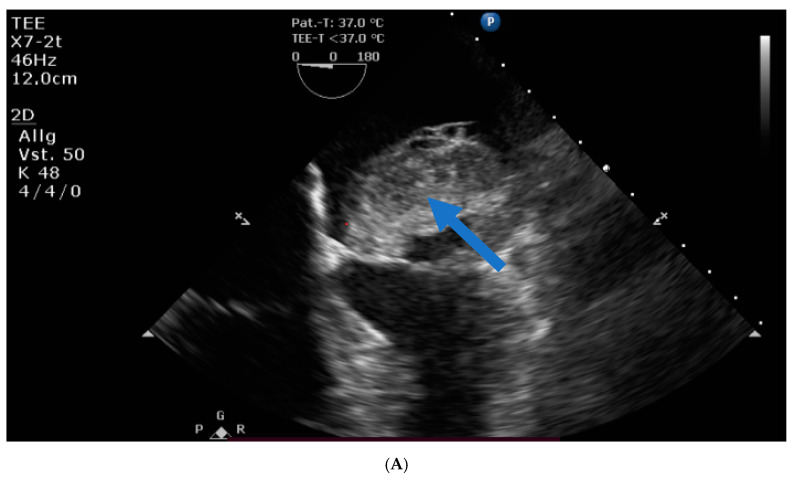

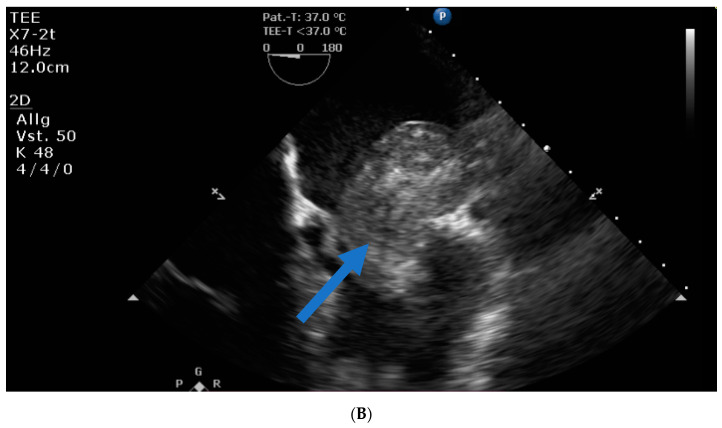
(**A**) Huge left atrial mass (arrow indicates cardiac mass). (**B**) Huge left atrial mass prolapsing into the mitral valve in diastole and thus obstructing the inflow of the left ventricle (arrow indicates cardiac mass).

Transthoracic and transesophageal echocardiography revealed a large mass (40 × 60 × 24 mm) in the left atrial lateral wall, extending to the mitral valve (Figure 1A,B). Both atria were severely dilatated. The left ventricle was normal in size with an ejection fraction of 55%, an apicolateral/midlateral hypokinesia and a mild diastolic dysfunction. The normal-sized right ventricle had impaired systolic function. A hemodynamically irrelevant pericardial effusion was present.

Sonography showed a small nodule in the left thyroid lobe.

The patient was referred to our tertiary cardiac surgery center for further treatment because of the tumor diagnosis. On the day of admission, the patient was in a reduced but stable cardiac condition. The previous findings of cardiac diagnostic imaging could be confirmed. Perioperative ECG showed tachycardic atrial fibrillation and right bundle branch block with left anterior fascicular block. Abdominal ultrasound, performed to exclude abdominal malignancy, was unremarkable; liver, spleen and kidneys were normal; and there was no free abdominal fluid.

A comprehensive laboratory profile revealed a thrombocytopenia of 55 × 10^3^/µL and a spontaneous INR of 1.28 but provided no evidence of the underlying cause.

The following day, the patient acutely decompensated and developed catecholamine-dependent cardiogenic shock. The emergency echo showed almost complete obstruction of the mitral valve inflow by the huge mass. Laboratory analysis revealed lactic acidosis and a further drop of thrombocytes to 23 × 10^3^/µL.

As a result, the patient underwent emergency cardiac surgery. Intraoperatively, the systolic function of the massively enlarged right ventricle was severely reduced. When the left atrium was opened, a large mass was seen, emanating from the left atrial appendage and obstructing the mitral valve orifice.

**Figure 2:**
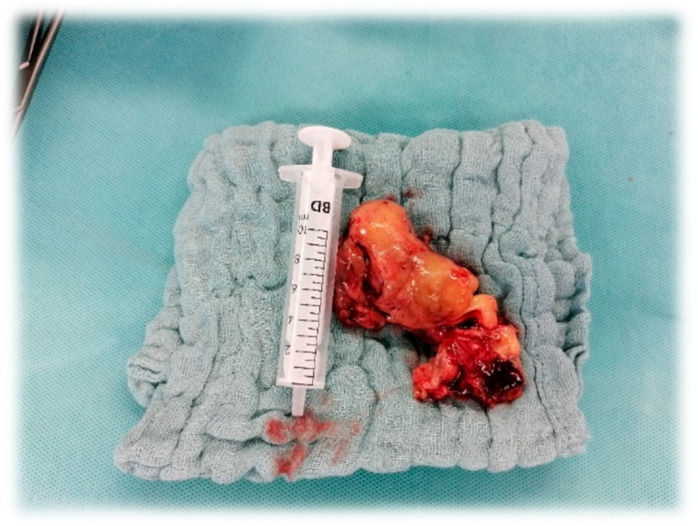
Left atrial tumor.

The mass was completely removed surgically (Figure 2). The mitral valve remained intact. After successful completion of the extracorporeal circulation, right ventricular pump function recovered and left ventricular contractility remained stable.

Unexpectedly, the patient developed acute right heart failure that was refractory to inotropic support and required extracorporeal membrane oxygenation (ECMO). Under central-ECMO (arterial cannula: aorta, venous cannula: left femoral vein) the inotropic support was reduced. After chest closure, the patient was transferred to the intensive care unit. Total aortic cross-clamp and CPB times were 43 min and 69 min, respectively.

Postoperatively, blood loss from the drains was severe. Laboratory tests revealed an activated clotting time (ACT) of 220 and a decreased platelet count. A second-look surgical intervention successfully controlled diffuse hemorrhaging from the operative side. Under ECMO support, the patient was transferred back to the intensive care unit with an open chest and without catecholamines. Thoracic closure was achieved with good ECMO function on postoperative day 3. At that time, a cranial CT ruled out active intracranial bleeding and cerebral metastasis.

On the sixth POD under ECMO support, a TTE revealed an LV with normal size, an ejection fraction of 45–50% and a mild apical hypokinesia. The aortic valve had a mild central insufficiency. The left atrium was enlarged. No mitral valve abnormalities were detected. The right ventricle was small, with a good contractility. No pericardial effusion was seen.

Under these conditions, ECMO support was reduced successfully, and explantation was indicated on the 12th POD. Intraoperatively, without mechanical ECMO support, a well-contractile LV and a well-pumping RV were noted. The chest was closed and the patient transferred to the intensive care unit on low doses of catecholamines.

Shortly thereafter, she developed again severe heart failure that was refractory to inotropic support.

**Figure 3:**
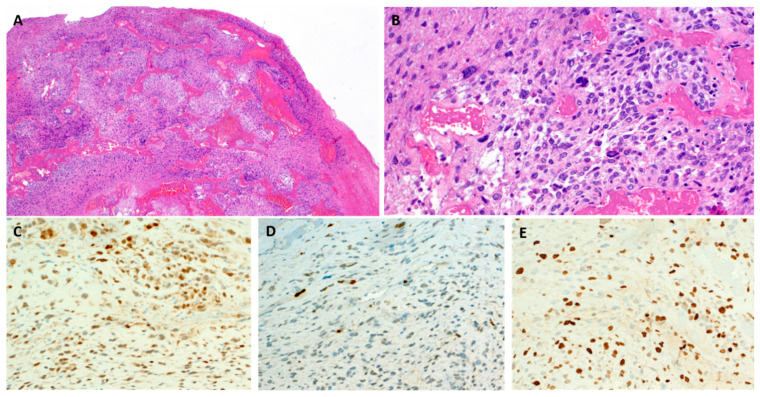
(**A**–**E**) Histological examination of left atrial tumor. (**A**,**B**) Undifferentiated neoplasm consisting of large, spindle-shaped pleomorphic cells arranged in solid aggregates and loose fascicles within an angiomyxoid stroma. Brisk mitotic activity with multiple atypical mitoses. (**C**) Immunohistochemical staining: expression of CDK4. (**D**) Immunohistochemical staining: variable expression of MDM2. (**E**) Immunohistochemical staining: KI67 labeled >50% of neoplastic cells.

**Figure 4:**
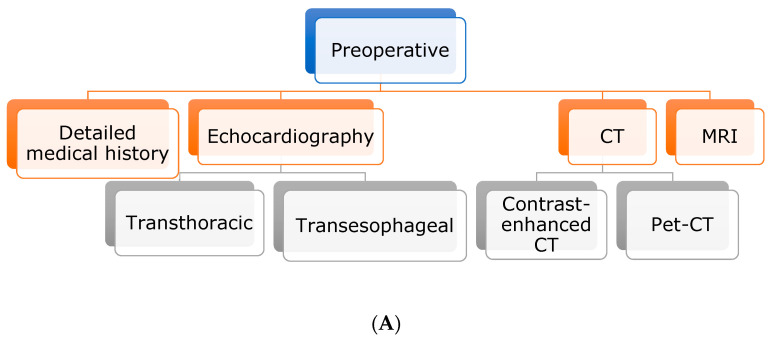

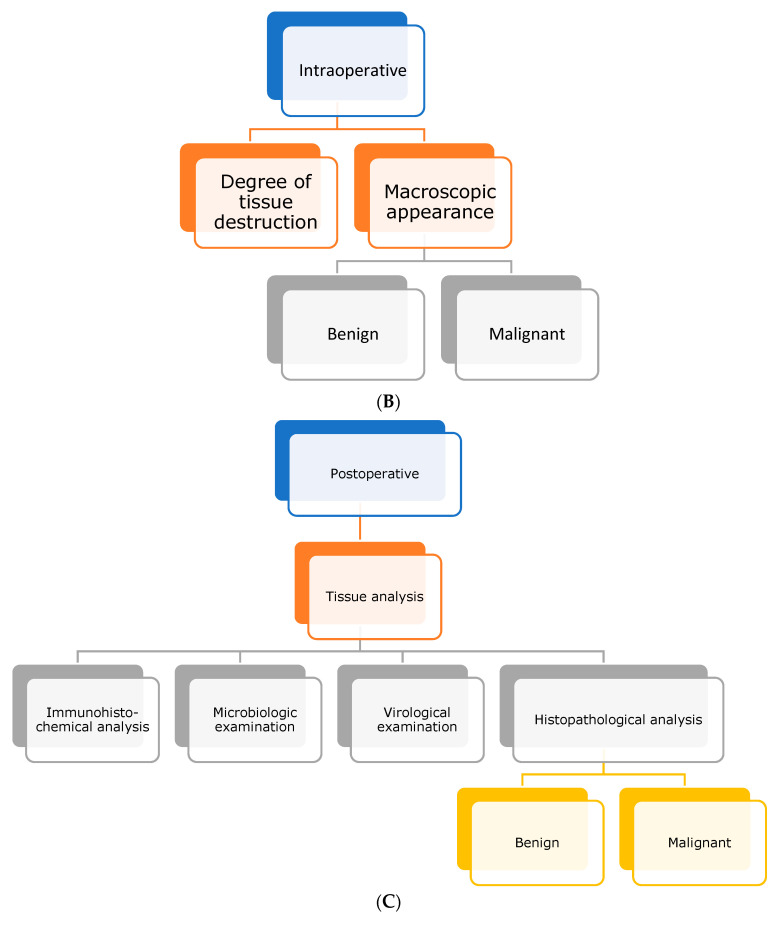

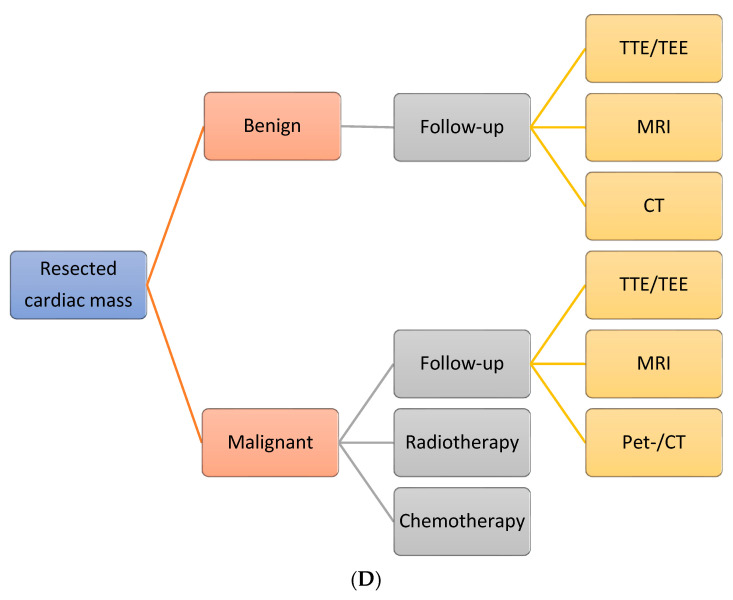
(**A**) Preoperative diagnostical steps of cardiac masses. (**B**) Intraoperative management of cardiac masses. (**C**) Postoperative diagnostic steps. (**D**) Postoperative treatment guide for cardiac masses.

In the meantime, the result of the pathologic–anatomical examination of the tissue samples taken intraoperatively (Figure 3A–E and Figure 4B,C) were available, which revealed the diagnosis of an undifferentiated pleomorphic cardiac sarcoma. Because of the resulting poor prognosis that could be deduced from this diagnosis and the exhausted therapeutic options, it was decided not to intensify the therapy, and the patient died of her devasting disease.

Pathologic gross examination revealed a fleshy soft exophytic and infiltrating mass, measuring 11.5 cm in aggregate. Histology showed an undifferentiated neoplasm composed of large spindled pleomorphic cells disposed into solid aggregates and loose fascicles within angiomyxoid stroma (Figure 3A,B). A brisk mitotic activity was noted with several atypical mitoses. The immunohistochemical staining revealed consistent expression of CDK4 (Figure 3C) and variable expression of MDM2 (Figure 3D) while other relevant markers tested negative (CD31, CD34, ERG, pankeratin AE1/AE3 and calretinin). KI67 highlighted >50% of the neoplastic cells (Figure 3E). Thus, the findings corresponded to an undifferentiated pleomorphic sarcoma with myxofibrosarcoma-like characteristics and CDK4 and MDM2 coexpression (so-called cardiac intimal sarcoma).

## 2. Discussion

We present a case in which a patient was diagnosed with a malignant cardiac tumor located in the left atrium, which ultimately led to the patient’s death. While many, but not all, cardiac masses on the left side of the heart are benign, a small subset of cases are malignant, mostly representing undifferentiated sarcomas with or without myxoid features. On the contrary, right-sided masses may suggest either metastatic disease or represent angiosarcomas [11]. Cardiac structures, which can appear as pseudotumors, are of differential diagnostic importance.

Unfortunately, patients with malignant cardiac tumors present at an already advanced stage in the majority of cases. The diagnosis of cardiac tumors can be challenging, and atrial tumors are often misdiagnosed by echo imaging as benign myxoma [8]. But if they are malignant, not properly recognized or treated too late, they are often fatal [6,8]. 

Primary cardiac tumors are rare and can be benign or malignant, occurring at an incidence of 0.00017–0.019% in autopsy. In the literature, malignant cardiac tumors are described in only 25%, while benign tumors occur in 75% [1]. Metastases in the heart are rare as well but more frequent than primary heart tumors [12,13]. 

Benign tumors mainly include myxomas, lipomas, fibroelastomas, rhabdomyomas, leiomyomas and fibromas. In the case of malignant cardiac tumors, different types also exist, e.g., angiosarcoma, rhabdomyosarcoma, leiomyosarcoma, liposarcoma, osteosarcoma, fibrosarcoma, malignant fibrous histiocytoma [1]. The most common malignant tumors are sarcomas. Pleomorphic tumors are exceptionally rare [6]. 

For a long time, particularly affected patients with benign cardiac tumors are asymptomatic. Malignant cardiac tumors, on the other hand, are characterized by a short history and a rapid course [14]. The typical age of manifestation is between 20 and 60 years. In three-fourths of cases, pulmonary, pleural, mediastinal or hepatic metastases are already present at the time of diagnosis [15,16]. 

The clinical symptoms depend on the tumor size, location and mobility.

Common symptoms are dyspnea, chest pain, syncope, fever, malaise and reduced general condition [5]. Well-known tumor complications are congestive heart failure, cardiac arrythmias, pulmonary embolism, valvular inflow obstruction as well as destruction of the ventricular inflow or outflow tract [2,3,4]. 

As it is not uncommon for tumor patients to experience thromboembolic events as an initial manifestation, a thorough diagnosis should be established in suspected patients with a thromboembolic event, and the source of embolism should be identified promptly.

During clinical examination, auscultation may reveal a characteristic early diastolic “tumor plop” [11]. Pulmonary congestion is a typical presentation of masses invading the left atrium.

Laboratory abnormalities are often unspecific but may include elevation in erythrocyte sedimentation rate, C-reactive protein and globulin levels and anemia.

For further diagnosis and differential diagnosis, the use of cardiovascular imaging is of utmost importance. Usually, the gold standard for evaluation of cardiac masses is transthoracic echocardiography, followed by transesophageal echocardiography and CT and/or MRI. If malignancy is suspected, PET-CT should be performed to rule out metastatic spread [6].

Definitive diagnosis, however, can only be achieved by pathohistological examination, including histoimmunological staining [7,8]. A wide variety of methods are available for obtaining tissue material, including catheter biopsy of the suspicious tissue or operative tumor excision. If a pericardial effusion is present, it can also be used for further pathological studies [8]. 

The treatment of cardiac masses is not clearly defined, and data for neoadjuvant chemotherapy or radiation therapy are scarce [3,10]. However, it is highly recommended to surgically remove all resectable tumor masses at an early stage for exact diagnosis and to prevent further complications or worsening of the patient’s condition [9]. This is particularly true if there are indications of malignancy, a risk of embolization or a mechanical complication of the heart valve. The indication for heart transplantation in malignant heart tumors is controversial [17]. However, there are also reports of favorable outcomes in primary malignant heart tumors after aggressive multimodality treatment [18].

After removal, depending on the pathologic diagnosis, these patients should continue in follow-up examinations to rule out both recurrence and metastasis.

In any case, surgical treatment of cardiac tumors should be performed in tertiary care centers, where arising complications can be managed.

In the case described, the patient presented with unspecific symptoms. The diagnosis of a left atrial tumor was made accidentally by echocardiography and confirmed by MRI. The acute symptoms of the cardiac decompensation could be attributed to the obstruction of the mitral valve orifice.

Due to the tumor-induced cardiogenic shock, emergency cardiac surgery was necessary. After successful tumor resection, pathological examination revealed a cardiac sarcoma. The clinical picture was so advanced that cytostatic polychemotherapy or radiation therapy was no longer considered, and the patient died 12 days postoperatively.

The case shows that early diagnosis of cardiac tumors can be crucial to initiate appropriate therapeutic measures and how late diagnosis can may adversely affect prognosis.

For diagnostic purposes, the full spectrum of sophisticated imaging methods should always be used to complete the actual diagnosis and to exclude other differential diagnoses. Therefore, it should always be taken into account that any cardiac mass has to be considered potentially malignant.

The definitive diagnosis can only be achieved by pathohistological and immunohistological examination [19]. In the present case the pathological examination revealed a highly malignant undifferentiated pleomorphic cardiac sarcoma, formerly called maligned fibrous histiocytoma**.** Most of these aggressive cardiac tumors, which have a predominance in women [20], are typically located in the left atrium, mimicking atrial myxoma or mitral valve stenosis. There is also the possibility of infiltration of the atrial septum or the left ventricle from there.

Most patients with cardiac sarcoma present at an already advanced stage, as in our presented case. Up to 80% of the cases have distant metastases at time of diagnosis [21].

In the present case, the cause of thrombocytopenia is not known, and various causes can be discussed. One explanation for the thrombocytopenia may be the tumor itself. Certain solid malignancies, or leukemia and lymphoma, can trigger paraneoplastic thrombocytopenia through immune-mediated mechanisms. Thrombocytopenia is a common complication among patients with solid tumors that predispose them to bleeding disorders resulting from several factors such as polymorphism and mutation in some transcription factors and cytokines involved in megakaryocytic maturation [22].

Furthermore, several studies highlighted the association between shock and thrombocytopenia, severe infection, trauma or blood loss. Under these conditions, platelets may be consumed or sequestered in damaged tissues. As a result, the circulating platelet count decreases, increasing the risk of bleeding. Particularly, patients treated with mechanical circulatory support and who have an adverse prognostic marker can suffer from thrombocytopenia [23]. 

To summarize, cardiac sarcomas result in rapid tumor growth and rapid death, and treatment options are very limited. The current literature describes survival rates as short as 9–10 months on average [5]. 

## 3. Conclusions

Perioperative transthoracic echocardiography described a cardiac mass (Figure 4A). Only pathological and immunohistological examinations demonstrated malignancy (Figure 4C). Any cardiac mass should be classified as potentially malignant. The full spectrum of diagnostics should be used to exclude other differential diagnoses and to narrow down the actual diagnosis. Tumors of the heart should be removed as soon as possible to prevent further complications or worsening of the patient’s condition. Patients should continue to be included in close follow-up examinations with regular imaging diagnostics to rule out both recurrence and metastasis (Figure 4D). In addition, consideration should be given to having operations on cardiac tumors performed at centers of maximum care, and the possibility of extracorporeal membrane oxygenation should also be considered. Early diagnosis of the disease is crucial for the prognosis.

## Data Availability

Data sharing is not applicable to this article as no datasets were generated or analyzed during the current study.
